# Epicatechin gallate and its analogues interact with sortase A and β-lactamase to suppress *Staphylococcus aureus* virulence

**DOI:** 10.3389/fcimb.2025.1537564

**Published:** 2025-03-25

**Authors:** Fei Teng, Lihui Wang, Jingyao Wen, Zizeng Tian, Guizhen Wang, Liping Peng

**Affiliations:** ^1^ Department of Respiratory Medicine, The First Hospital of Jilin University, Changchun, China; ^2^ College of Biological and Food Engineering, Jilin Engineering Normal University, Changchun, China

**Keywords:** ECG, sortase A, β-lactamase, biofilm, persisters, *Staphylococcus aureus*

## Abstract

*Staphylococcus aureus* sortase A can anchor virulence proteins, which are responsible for bacterial adhesion, biofilm formation, and inflammation, to the cell membrane surface. The ability of β-lactam antibiotics to combat *S. aureus* infections is limited by the presence of β-lactamases in this pathogen. In this study, we determined that epicatechin gallate (ECG) and its analogues inhibited the transpeptidase activity of sortase A by interacting with it directly, and the biofilm formation and adhesion abilities of the bacterium decreased after treatment with ECG and its analogues. Additionally, ECG bound to β-lactamase and reduced its ability to hydrolyze nitrocefin. Furthermore, ECG synergized with ampicillin (Amp), enhancing its bactericidal effects and inhibiting the formation of persisters. ECG did not affect the expression of sortase A or β-lactamase but significantly alleviated the cytotoxicity of *S. aureus* USA300. ECG alone or combined with Amp *in vivo* improved the survival of mice infected with *S. aureus* USA300, alleviated pathological tissue damage and pulmonary edema, and reduced the extent of inflammation and level of colonization. The results of this study indicate that the active ingredients of green tea, especially ECG, have the potential to be developed as anti-*S. aureus* infection agents.

## Introduction

For successful infection, *Staphylococcus aureus* (*S. aureus*) must adhere to and colonize the host (Ronner et al., 2014; Foster, 2019), and this process is aided by adhesins covalently anchored to the cell wall by sortase A (Foster et al., 2014; Foster, 2019; Alharthi et al., 2021). Sortase A inhibitors significantly reduce bacterial adherence to cells (Liu et al., 2015; Bozhkova et al., 2022; Shulga and Kudryavtsev, 2022). In addition, sortase A can anchor many other surface proteins to the cell wall, which could improve bacterial pathogenicity (Jonsson et al., 2003; Weiss et al., 2004) through mechanisms such as affecting biofilm formation (Kim et al., 2018; Xu et al., 2024) and promoting *S. aureus* evasion of the host immune system (Weiss et al., 2004). Some inhibitors targeting sortase A have been reported to reduce bacterial adherence to host cells and biofilm formation, thereby increasing the survival rate of model animals with *S. aureus* infection (Song et al., 2022; Su et al., 2022; Jiang et al., 2023). The number of virulence proteins anchored to the bacterial cell wall decreases significantly upon sortase A inactivation, which results in a reduction in *S. aureus* pathogenicity. Therefore, sortase A is an ideal target for developing inhibitors of *S. aureus* infection (Cascioferro et al., 2014; Cascioferro et al., 2015; Alharthi et al., 2021).

*S. aureus* β-lactamase can hydrolyze almost all β-lactam antibiotics, including the recently developed cephalosporins and all carbapenem antibiotics, known as the “last antibiotics”, which imposes a considerable challenge for the clinical treatment of *S. aureus* infection (Mossman et al., 2022; George et al., 2023); however, the development of new antibiotics is slow. Therefore, restoring bacterial sensitivity to current antibiotics is an effective strategy to alleviate resistance. Several compounds have been shown to restore bacterial susceptibility to antibiotics by targeting β-lactamases, indicating that restoring bacterial sensitivity to this class of antibiotics is feasible by inhibiting β-lactamase activity.

Catechins are active phenolic substances that are mainly found in green tea, including the main active components epicatechin gallate (ECG), catechin (C), epicatechin (EC), epigallocatechin (EGC), and epigallocatechin gallate (EGCG) (Kochman et al., 2020; Musial et al., 2020). These compounds have considerable biological functions, such as antiviral, antitumoral, antioxidant, and antithrombotic effects (Ide et al., 2016; Chen et al., 2021; Chen et al., 2022). EGCG has been reported to affect *Streptococcus pneumoniae* virulence by inactivating sortase A (Song et al., 2017), but compounds that act against both β-lactamase and sortase A simultaneously to suppress *S. aureus* virulence have not been reported. In this study, we discovered that the abovementioned components of green tea inhibited *S. aureus* USA300 infection by interacting with β-lactamase and sortase A. On the one hand, these compounds inhibited biofilm formation and bacterial adhesion by inhibiting sortase A. On the other hand, ECG, as a representative, restored the susceptibility of *S. aureus* USA300 to β-lactam antibiotics and protected mice from *S. aureus* USA300 infection.

## Materials and methods

### Reagents, strains, and growth conditions

The small molecules used in this study were obtained from Chengdu Purechem-Standard Co., Ltd. Penicillin G (PG), ampicillin (Amp), ceftriaxone sodium (Cef), kanamycin, and nitrocefin were purchased from Dalian Meilun Biotechnology Co., Ltd. Dulbecco’s modified Eagle’s medium (DMEM), fetal bovine serum (FBS), trypsin (0.25%), and penicillin−streptomycin solution were purchased from Beijing Solarbio Science & Technology Co., Ltd. The substrate peptide of sortase A (Dabcyl-QALPETGEE-Edans) was purchased from GL Biochem (Shanghai), Ltd. *S. aureus* USA300, which expresses β-lactamase, was stored in our laboratory and cultured in Luria−Bertani (LB) media at 37°C with shaking or under static conditions.

### Protein preparation

The *S. aureus* sortase A gene was cloned and inserted into the pET-28a (+) vector, which was subsequently transformed into *Escherichia coli* BL21 (DE3) (Beyotime, Shanghai, China). The *E. coli* was cultured in LB medium supplemented with 0.2 mM isopropyl-beta-D-thiogalactopyranoside (Beyotime, Shanghai, China) at 18°C for 18 h. Then, the bacteria were harvested and lysed to obtain the supernatant. The supernatant was incubated with Ni-NTA agarose resin, and the purified protein was obtained after elution with 250 mM imidazole. To construct the sortase A mutants, primers designed for specific mutations (V168A, K173A, and L169A) were obtained using the QuikChange Site-Directed Mutagenesis Kit. The pET28a plasmid carrying the sortase A gene was used as a template, and the polymerase chain reaction (PCR) products were introduced into *E. coli* BL21 (DE3) after treatment with *DpnI* (Beyotime, Shanghai, China). The mutated proteins were obtained via the same purification method described above. Cloning, expression, and purification of β-lactamase and its mutants (Y96A, I158A, and I230A) were performed via the same methods. The mutants of sortase A and β-lactamase were used to identify the residues critical for interaction with the compounds. The primers used for this assay are shown in **Table 1**.

**Table 1:** The primers used for clone and site-directed mutagenesis.

Primer name	Oligonucleotide (5'-3')
Sortase A-F	CTGGGATCCCAAGCTAAACCTCAAATT
Sortase A-R	CTGGTCGACTTATTTGACTTCTGTAGC
β-lactamase-F	CTGGGATCCGCCAAAGAGTTAAATGATTTA
β-lactamase-R	CTGGTCGACTTAAAATTCCTTCATTAC
Sortase A V168A-F	CTACAGATGTAGGAGCGCTAGATGAACAAAAAG
Sortase A V168A-R	CTTTTTGTTCATCTAGCGCTCCTACATCTGTAG
Sortase A K173A-F	CTAGATGAACAAGCGGGTAAAGATAAAC
Sortase A K173A-R	GTTTATCTTTACCCGCTTGTTCATCTAG
Sortase A L169A-F	GATGTAGGAGTTGCGGATGAACAAAAAG
Sortase A L169A-R	CTTTTTGTTCATCCGCAACTCCTACATC
β-lactamase Y96A-F	GATATAGTTGCTGCGTCTCCTATTTTAG
β-lactamase Y96A-R	CTAAAATAGGAGACGCAGCAACTATATC
β-lactamase I158A-F	GTTAGATATGAGGCGGAATTAAATTAC
β-lactamase I158A-R	GTAATTTAATTCCGCCTCATATCTAAC
β-lactamase I230A-F	GTGGTCAAGCAGCGACATATGCTTC
β-lactamase I230A-R	GAAGCATATGTCGCTGCTTGACCAC

The underlines represent the mutation sites or cleavage sites.

### Inhibition of sortase A activity

The sortase A protein (10 µg) was coincubated with various concentrations of ECG and its analogues (0, 8, 16, and 32 µg/mL) at 37°C for 0.5 h. The substrate peptide (10 µg, final volume of 100 µL) was subsequently added, and the samples were incubated for 1 h in the dark. Then, the fluorescence intensity was detected by using a microplate reader (excitation 490 nm, emission 520 nm), and the inhibitory effects of ECG and its analogues against sortase A were evaluated via a previously described method (Song et al., 2017). The inhibitory effects of ECG against the mutants of sortase A (V168A, K173A and L169A) were determined via the same method.

### Computational biology

AutoDock Vina software (Trott and Olson, 2010) was used to perform docking calculations after the compounds (set as the ligands) were docked into proteins (β-lactamase PDB ID: 6WGR; sortase A PDB ID: 6R1V) with AutoDock Tools on the basis of methods described previously (Gao et al., 2020). Molecular dynamics simulations of the candidates were carried out using GROMACS 2020.6 and previously reported methods (Liu TT. et al., 2019; Gao et al., 2020). Briefly, RESP charges were assigned to the ligands using Gaussian and amber, and topology files of the ligands were obtained by using AmberTools. The Amber 14sb force field and SPC/E water model were also used. The root mean square deviation (RMSD) was used to evaluate the equilibrium of the system, and key protein residues involved in the interactions with the ligands were identified via the molecular mechanics Poisson−Boltzmann surface area (MMPBSA) method.

### Biofilm inhibition

Different concentrations of ECG and its analogues (0, 8, and 32 µg/mL) were used to treat *S. aureus* USA300, which were seeded into 96-well plates (5 × 10^6^ CFUs/well). The samples were cultured at 37°C until static. At 24 h later, the medium was removed, and the samples were washed with sterile phosphate-buffered saline (PBS) and stained with 0.1% crystal violet. The optical density at 570 nm (OD_570_) of each well was measured after adding 33% glacial acetic acid to dissolve the solids to evaluate the biofilm formation inhibitory effects of the compounds (Grossman et al., 2021). The free LB medium was defined as negative control (NC).

### Inhibition of β-lactamase activity

*S. aureus* USA300 β-lactamase protein activity inhibition assays were performed according to methods reported previously (Zhou et al., 2020). Briefly, different concentrations of ECG and its derivatives (0, 32, 64, and 128 µg/mL) were used to treat the purified β-lactamase protein (5 µg) for 30 min at 37°C, and nitrocefin (5 µg) was added for the final 10 min of incubation. The OD_492_ of each well was subsequently measured to determine whether these compounds affect the activity of β-lactamase (Zhou et al., 2020). The inhibitory effects of ECG against the β-lactamase mutants (Y96A, I158A, and I230A) were determined via the same method.

### Analysis of the antimicrobial properties

The minimum inhibitory concentrations (MICs) of PG, Amp, Cef, and kanamycin combined with or without the test compounds were determined on the basis of Clinical and Laboratory Standards Institute (CLSI) methods. Briefly, LB culture medium containing a series of concentrations of PG, Cef, Amp, or kanamycin (0–256 µg/mL) and ECG derivatives (0–32 µg/mL) was added to a 96-well plate, followed by the addition of *S. aureus* USA300 strains (5 × 10^5^ CFUs/mL). Samples were cultured at 37°C for 24 h, and the MIC values were defined as the concentration of compound at which there was no visible bacteria growth. The antimicrobial properties of the ECG and its analogues and their synergistic effects with PG, Cef, Amp, and kanamycin were analyzed on the basis of the MIC values (Guo et al., 2024).

### Time-dependent bacterial killing effects and analysis of persisters

On the basis of the synergistic effect between the ECG and its analogues and the tested antibiotics, Amp combined with ECG (32 µg/mL) was used to carry out a time-dependent bacterial killing assay. *S. aureus* USA300 in the logarithmic growth phase (approximately 2 × 10^6^ CFUs/mL) was treated with ECG (32 µg/mL), Amp (64 µg/mL), or their combination and cultured with shaking. Samples from each group were collected every 2 h and plated on LB agar medium after dilution. The colonies were analyzed to evaluate the ability of ECG to improve the bactericidal ability of Amp (Liu S. et al., 2019). The persister assay was performed on the basis of a previously described method with some modifications (Keren et al., 2004). Briefly, *S. aureus* USA300 was treated with 10 µg/mL gentamicin, then ECG (32 µg/mL) was added, and the mixture was cultured with shaking. Finally, bacterial colonies were harvested at the indicated time points to analyze the formation of persisters (Keren et al., 2004).

### Cell culture and assays

Mouse mononuclear macrophages (RAW264.7 cells) and human lung cancer epithelial cells (A549 cells) were cultured in DMEM supplemented with 10% FBS at 37°C with 5% CO_2_. The RAW264.7 cells seeded in 96-well plates (2 × 10^4^ cells/well) were treated with *S. aureus* USA300 at a multiplicity of infection (MOI) of 60, after different concentrations of ECG (0, 16, or 32 µg/mL) were added, and the samples were cocultured for 5 h. Then, the samples were centrifuged (1,000 rpm for 10 min), the supernatant was added to an equal volume of lactate dehydrogenase (LDH) reagent (Solarbio, Beijing, China), and the mixture was incubated in the dark for 30 min. The level of LDH in each sample was determined by measuring the OD_490_. After having been washed with sterile PBS, the cells were treated with ethidium bromide (EB, 50 µg/mL) to observe the inhibitory effect of ECG on the toxicity of *S. aureus* USA300 by using fluorescence microscopy (OLYMPUS, IX83). The cytotoxicities of the tested compounds were determined by detecting the LDH levels. For the adhesion assay, A549 cells in 24-well plates (1 × 10^5^ cells/well) were treated with *S. aureus* USA300 (MOI = 30) containing ECG (0, 8, or 32 µg/mL). At 1 h later, the medium was discarded, the cells were washed with sterile PBS, and the samples were plated onto LB agar medium after dilution. The number of colonies was used to assess the inhibitory effect of ECG on bacterial adhesion.

### Protein expression and secretion analysis

*S. aureus* USA300 was cocultured with different concentrations of ECG (0, 16, or 32 µg/mL) for 8 h. The bacteria and supernatants were subsequently collected separately after centrifugation (12,000 rpm for 5 min). The expression levels of sortase A and β-lactamase in the bacteria were detected by western blotting. The secretion of β-lactamase into the supernatant was evaluated via Coomassie brilliant blue staining. The antibodies used in these experiments were stored in our laboratory (mouse sortase A, 1:1,000; mouse β-lactamase, 1:1,000; goat anti-mouse secondary antibodies, 1:10,000).

### Animal infection model

Male C57BL/6J mice (weighing approximately 20 g) were purchased from Liaoning Changsheng Biotechnology Co., Ltd. All animal experiments were performed in accordance with the requirements of Jilin University. The mice had free access to food and water. The mice were intranasally inoculated with 30 μL of *S. aureus* USA300 suspension (5 × 10^8^ CFUs) to establish a pneumonia model. At 2 h later, ECG (50 mg/kg), Amp (50 mg/kg), or their combination was administered to the infected mice. Mice in the positive control (PC) group were infected with *S. aureus* USA300 and administered an equal volume of solvent instead of compound, and the mice in the negative control (NC) group were administered an equal volume of sterile PBS only. A total of 10 mice were assigned to each group for the survival assay, and the survival status of the mice was observed every 12 h. For other analyses, each group contained eight mice. After 48 hours of treatment, the mice were anaesthetized by injection of pentobarbital sodium (50 mg/kg) and then euthanized by cervical dislocation. The lung tissues of the mice were harvested, and pathological damage was observed after haematoxylin−eosin staining. Additionally, the tissue bacterial load was analyzed after homogenization and culture on LB agar, the ratio of the weight of each lung before and after drying was analyzed, and the levels of inflammatory factors in the alveolar lavage fluid was detected via enzyme-linked immunosorbent assays (ELISAs; Sangon Biotech, Shanghai, China).

### Statistical analysis

The data are presented as the means with the standard deviations (SDs) or standard errors of the means (SEMs) from three independent experiments. Statistical analysis was performed via the unpaired *t test* in GraphPad Prism 9.5.0. Statistical significance was defined as p ≤ 0.05.

## Results

### ECG and its analogues inhibit the activity of sortase A

**Figure 1:**
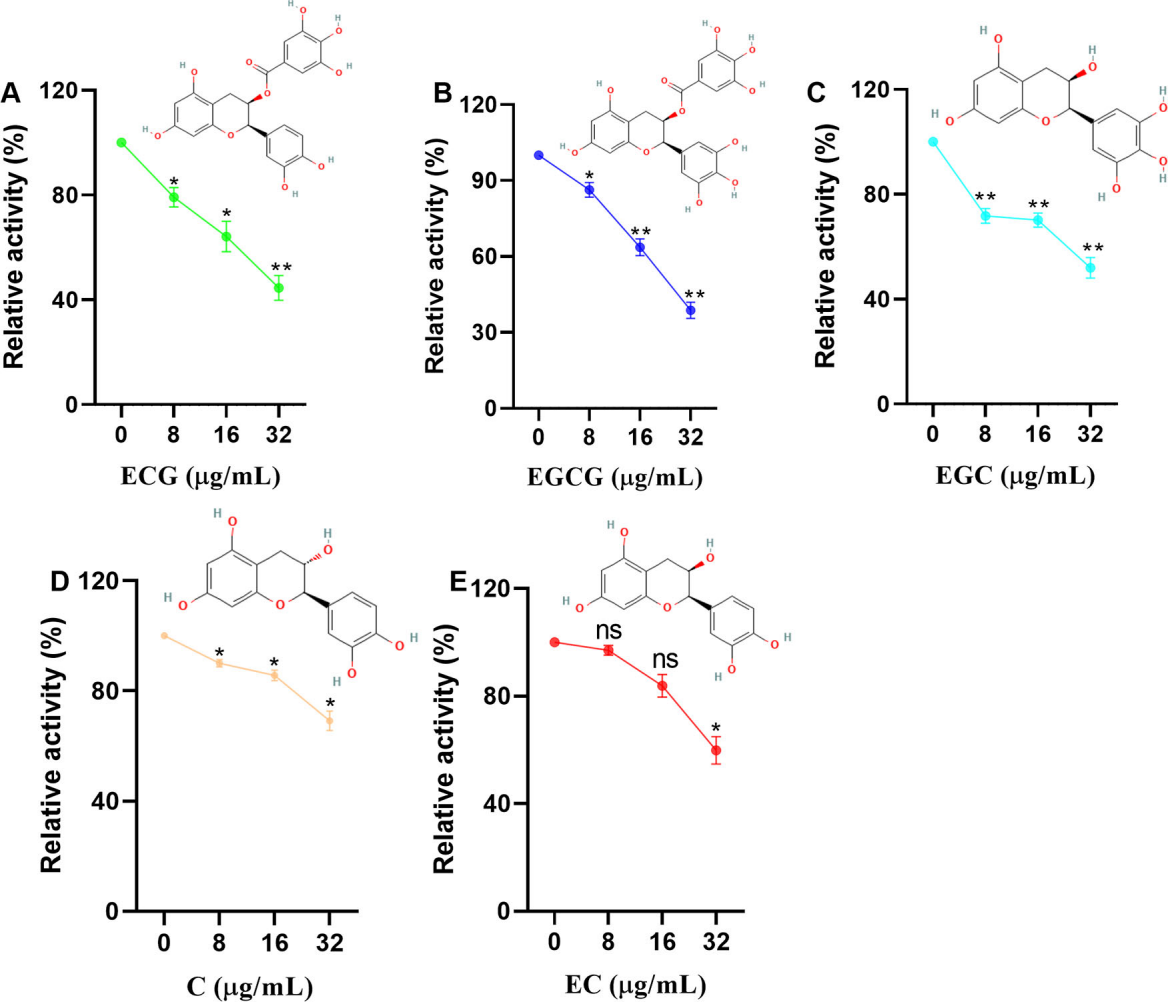
ECG and its analogues inhibit the activity of sortase A. Relative activities of sortase A after treatment with different concentrations of ECG **(A)**, EGCG **(B)**, EGC **(C)**, C **(D)**, and EC **(E)**. The sortase A protein was coincubated with different concentrations of ECG and its analogues at 37°C for 30 min, and then the substrate peptide was added for an additional 1 h of incubation. The fluorescence intensity (excitation 490 nm, emission 520 nm) was measured by using a microplate reader. The relative activity of sortase A was calculated by using the following formula: (S_1_/S_0_) × 100, where S_1_ or S_0_ represents the fluorescence value of samples treated with or without tested compounds. Data are shown as means with SEMs, *n* = 3; ns indicates not significant, * indicates *p* ≤ 0.05, and ** indicates *p* ≤ 0.01.

The relative activity of sortase A was defined as 100% at a test compound concentration of 0 µg/mL. Sortase A activity was reduced to 79.11%, 64.14% and 44.52% upon treatment with 8, 16 and 32 µg/mL ECG, respectively (**Figure 1A**). Similarly, reductions in sortase A activity were observed after treatment with 8, 16 and 32 µg/mL EGCG (13.68%, 36.35% and 61.22%, respectively) (**Figure 1B**), EGC (28.23%, 29.87% and 48.07%, respectively) (**Figure 1C**), C (10%, 14.32% and 30.88%, respectively) (**Figure 1D**) and EC (2.94%, 16.12% and 40.14%, respectively) (**Figure 1E**). These results showed that ECG and its analogues could significantly inhibit the activity of sortase A and that the gallic acid moiety played a role in this activity.

### ECG and its analogues show excellent affinity for *S. aureus* sortase A

**Figure 2:**
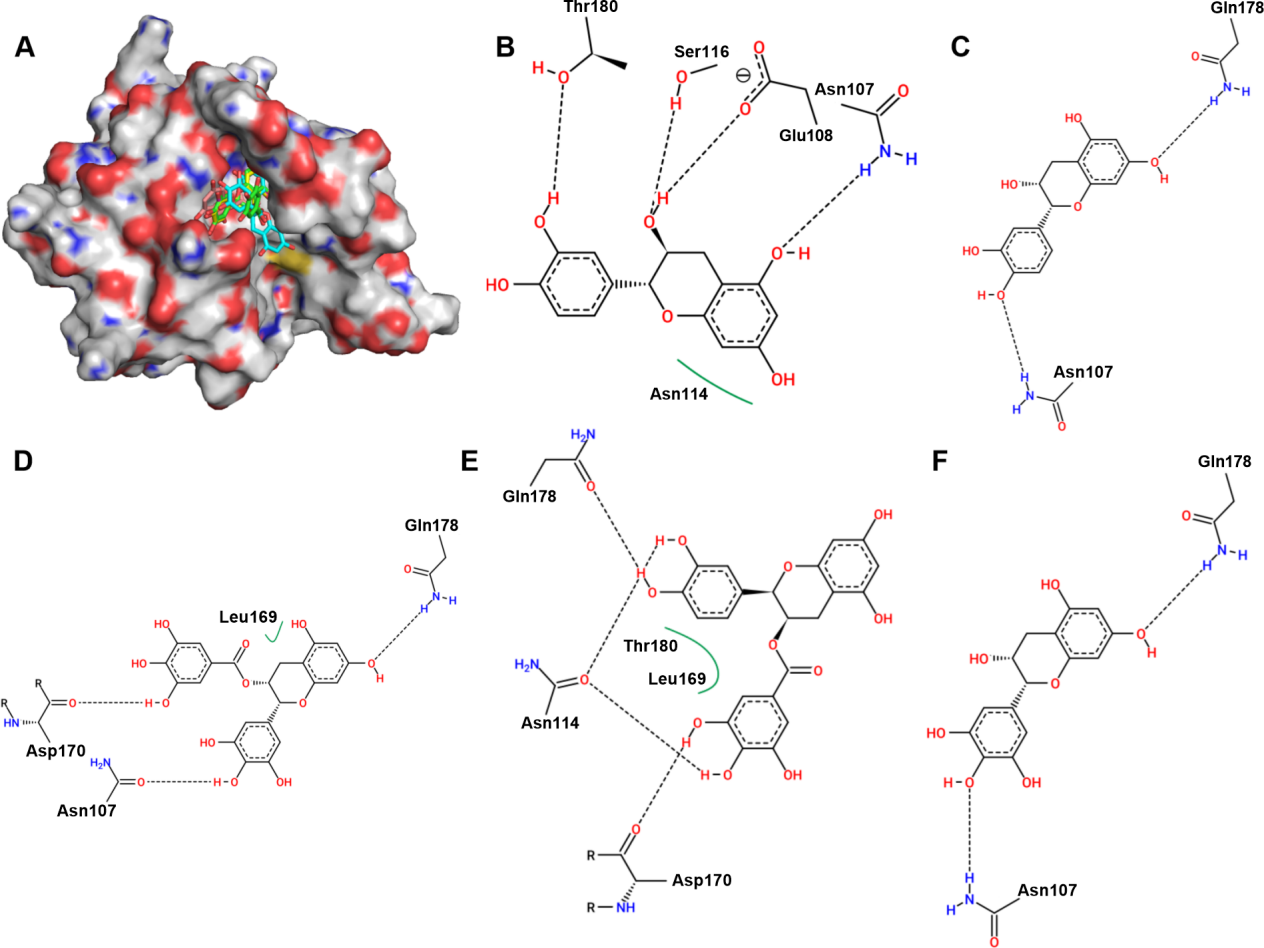
Binding mode of ECG and its analogues to sortase A and analysis of the potential binding sites. **(A)** Binding mode of ECG and its analogues to sortase A. Potential interaction sites of sortase A with C **(B)**, EC **(C)**, EGCG **(D)**, ECG **(E)**, and EGC **(F)**. Hydrogen atoms and charges were added to sortase A (PDB ID: 6R1V) and ECG and its analogues using AutoDock Tools, and AutoDock Vina was used to perform docking. Potential binding sites were analyzed using Proteins Plus.

Molecular docking was carried out, and these compounds were found to bind to the catalytic pocket of sortase A (**Figure 2A**). The binding energies of ECG, EGCG, EGC, EC, C to sortase A were -7.2, -7.0, -6.7, -6.97 and -6.9 kcal/mol, respectively. Analysis of the sortase A binding site revealed that Thr180, Ser116, Glu108, Asn107 and Asn114 are involved in the binding of EC to sortase A (**Figure 2B**); that Gln178 and Asn107 of sortase A interact with C (**Figure 2C**); that EGCG interacts with residues Gln178, Leu169, Asp170 and Asn107 of sortase A (**Figure 2D**); and that residues Gln178, Asn114, Leu169, Thr180, Asp170 (**Figure 2E**), Asn107 and Gln178 are involved in the interaction between EGC and sortase A (**Figure 2F**). These results indicate that the residues involved in the binding of each compound were not exactly the same even though all of the interacting residues were located in the same catalytic pocket.

### Residues Val168, Lys173, and Leu169 are critical for compound binding

**Figure 3:**
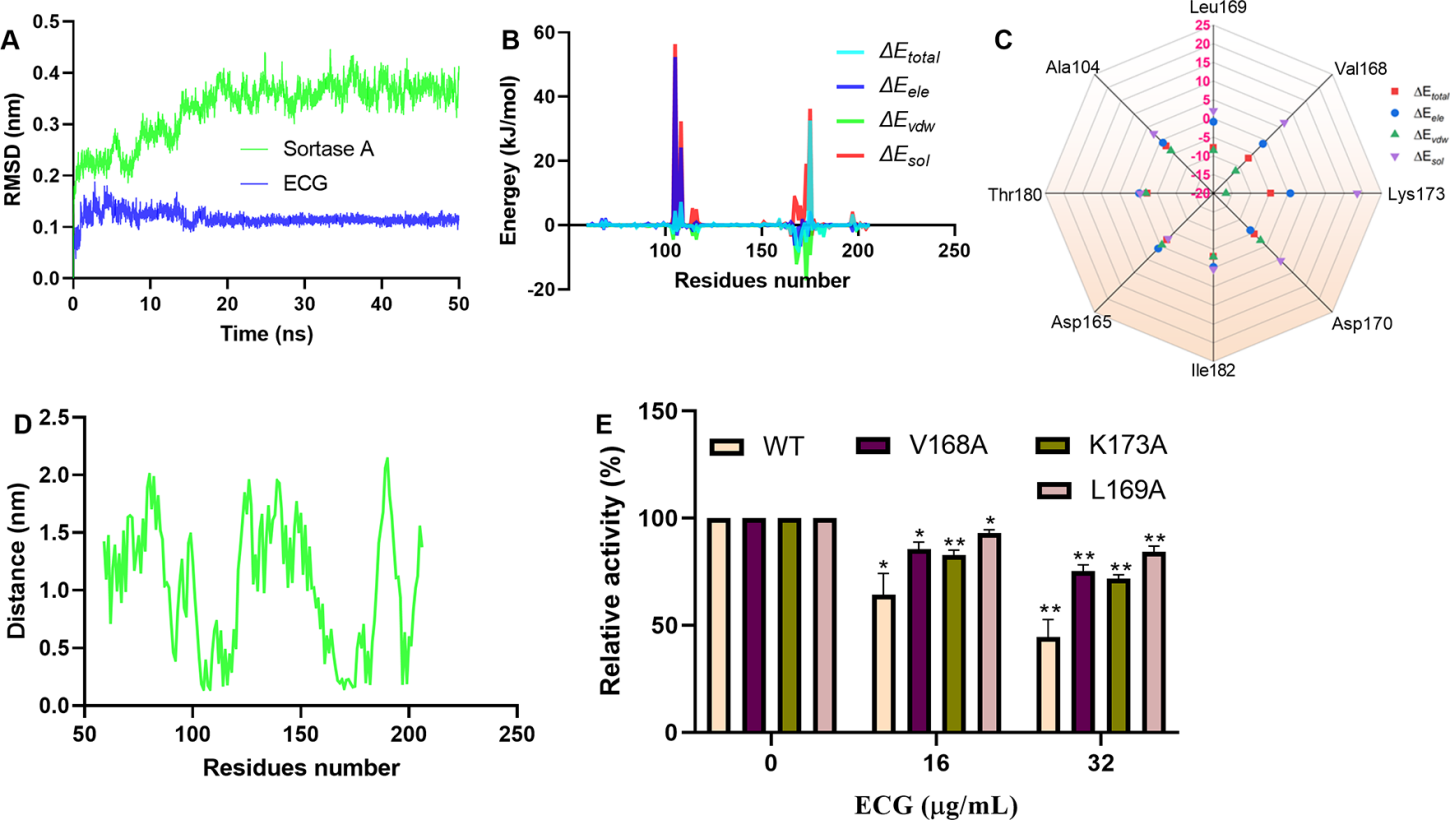
Identification of critical residues involved in ECG binding to sortase A. **(A)** RMSD values from the simulations. Contribution of each residue to the binding energy **(B)** and the residues with greater contributions **(C)**. **(D)** Distances between sortase A residues and ECG. **(E)** Inhibitory effects of ECG against sortase A and its mutants. GROMACS version 2020.6 was used to perform molecular dynamics simulations using the amber 14SB force field and SPC/E water model. The MMPBSA method was used to calculate the binding free energies. Data are shown as means with SDs, *n* = 3; * indicates *p* ≤ 0.05 and ** indicates *p* ≤ 0.01.

The RMSD values of sortase A and ECG fluctuated around 0.38 nm and 0.1 nm, indicating that sortase A and the ECG complex reached an equilibrium state during the molecular dynamics simulations (**Figure 3A**). Analysis of the binding free energies from the molecular dynamics simulations revealed that electrostatic interactions (ele), van der Waals forces (vdw) and solvation energy (sol) participated in compound binding (**Figure 3B**). Residue energy decomposition analysis revealed that Val168, Leu169, Ala104, Thr180, Asp165, Ile182, Asp170 and Lys173 have greater contributions to the binding energy (**Figure 3C**), which was confirmed by the closer proximity of these residues to ECG (**Figure 3D**). Site-directed mutagenesis was performed to construct the V168A, K173A and L169A mutants, and the inhibitory effects of ECG on these mutants were significantly reduced compared with inhibition of the WT protein (**Figure 3E**), indicating that these residues are important for ECG and sortase A binding.

### ECG and its analogues inhibit *S. aureus* USA300 biofilm formation

**Figure 4:**
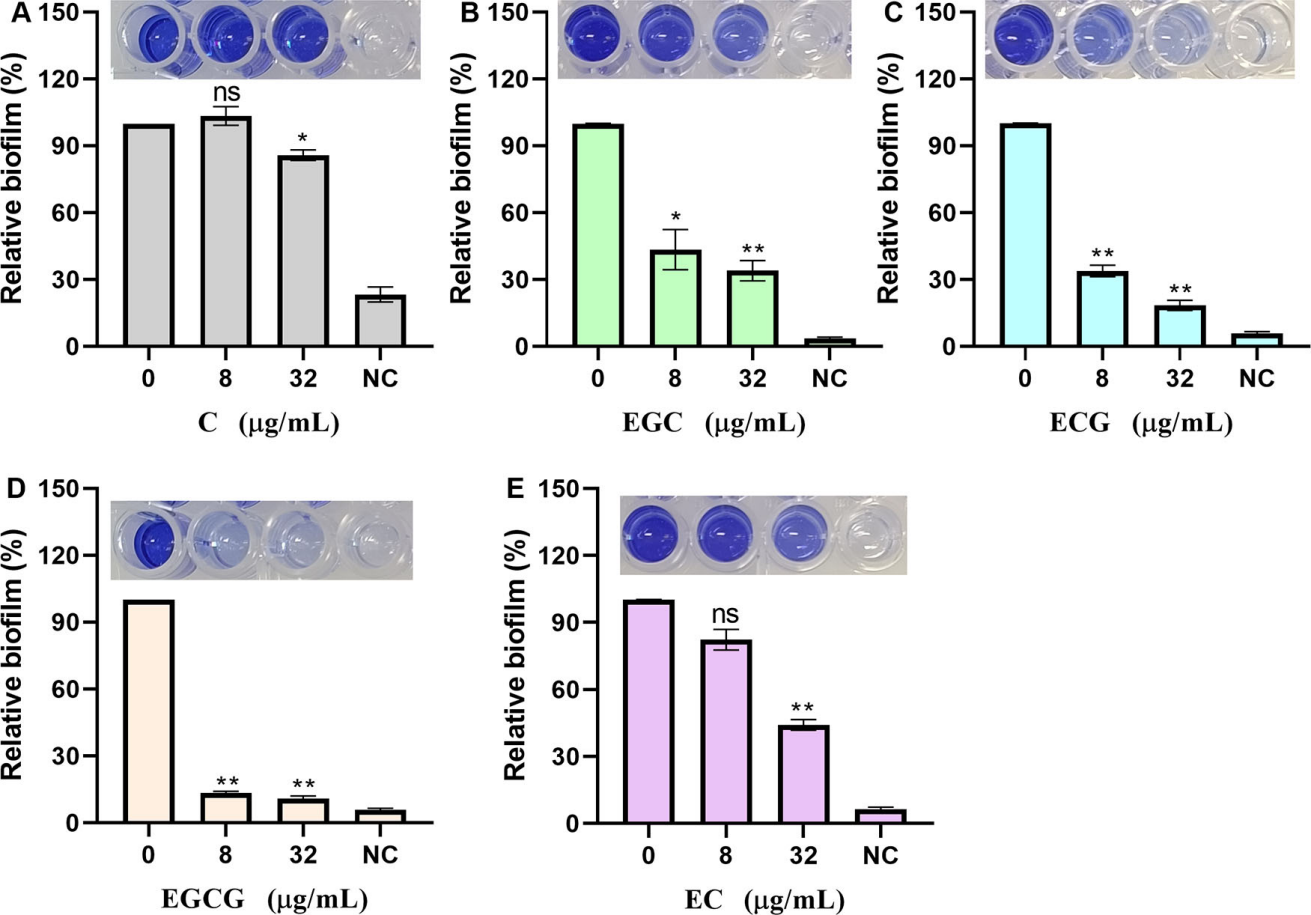
ECG and its analogues inhibit the biofilm formation of *S. aureus* USA300. Relative biofilm formation of *S. aureus* USA300 treated with different concentrations of C **(A)**, EGC **(B)**, ECG **(C)**, EGCG **(D)**, and EC **(E)**. *S. aureus* USA300 was cocultured with different concentrations of ECG and its analogues at 37°C for 24 h, after which the samples were stained with 0.1% crystal violet, washed, and fixed, and the OD_570_ was finally measured after treatment with 33% glacial acetic acid. NC indicates negative control. Data are shown as means with SEMs, *n* = 3; ns indicates not significant, * indicates *p* ≤ 0.05, and ** indicates *p* ≤ 0.01.

sortase A is closely related to biofilm formation. Here, we quantitatively analyzed the effects of ECG and its analogues on *S. aureus* USA300 biofilm formation via crystal violet staining. *S. aureus* USA300 biofilm formation was defined as 100% in the absence of test compounds. When the bacteria were treated with 8 or 32 µg/mL C, biofilm formation was 103.40% and 85.84%, respectively (**Figure 4A**). Similarly, biofilm formation was reduced to 43.48% and 34.83% when the bacteria were treated with 8 and 32 µg/mL EGC, respectively (**Figure 4B**), and these values were 33.83% and 18.44%, respectively, upon treatment with ECG (**Figure 4C**); 13.26% and 10.92%, respectively, upon treatment with EGCG (**Figure 4D**); and 82.33% and 44.19%, respectively, upon treatment with EC (**Figure 4E**). These results showed that ECG and its analogues inhibited *S. aureus* USA300 biofilm formation to different degrees.

### ECG and its analogues reduce the adhesion of *S. aureus* USA300 to lung epithelial cells

**Figure 5:**
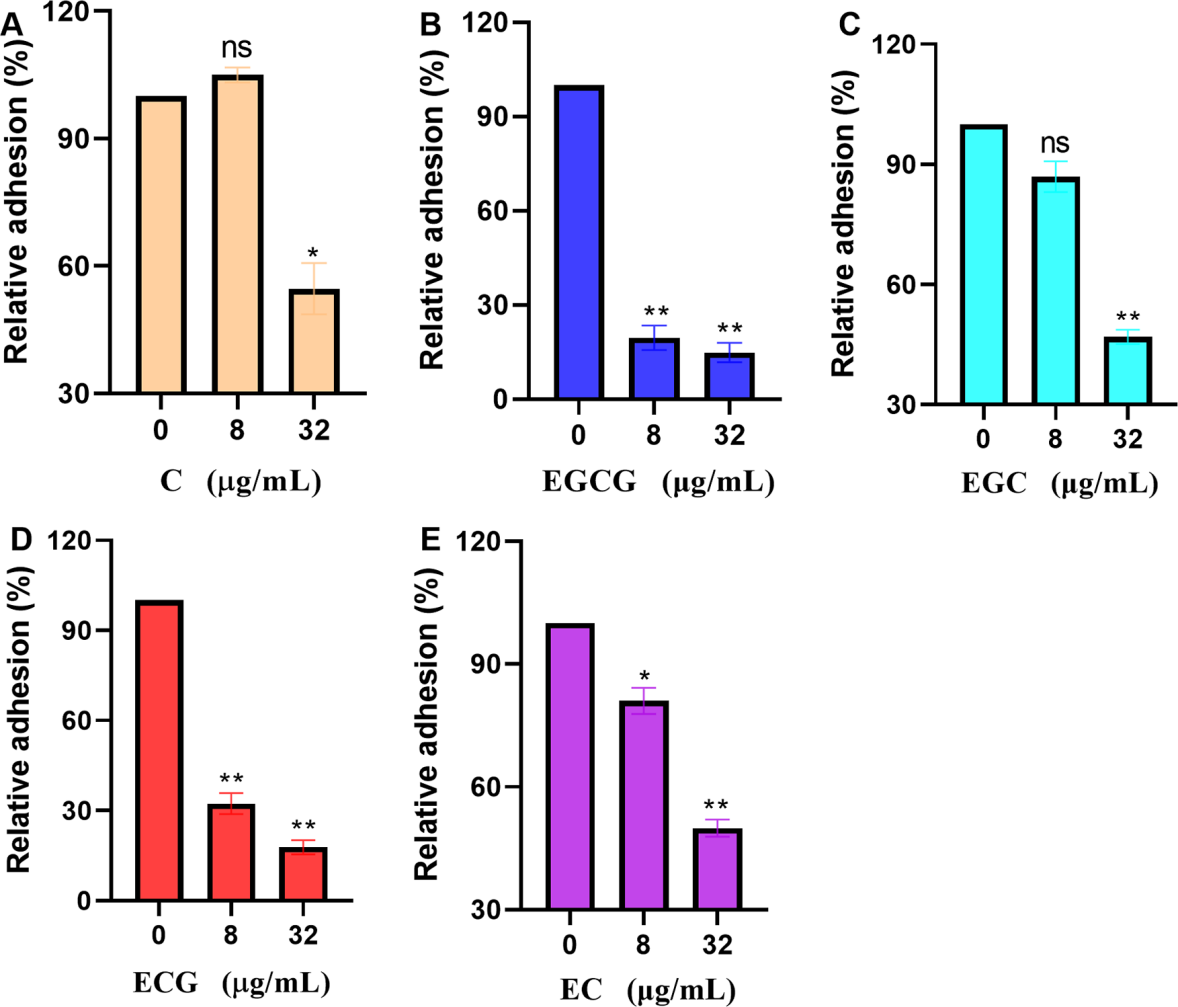
ECG and its analogues reduce the adhesion of *S. aureus* USA300 to lung epithelial cells. Relative adhesion of *S. aureus* USA300 to A549 cells after treatment with different concentrations of C **(A)**, EGCG **(B)**, EGC **(C)**, ECG **(D)**, and EC **(E)**. Human lung cancer epithelial A549 cells in 24-well plates (1 × 10^5^ cells/well) were treated with *S. aureus* USA300 (MOI = 30) and different concentrations of the test compounds for 1 (h). Then, the cells were harvested after discarding the medium, washed with sterile PBS, diluted, plated onto LB agar in equal volumes, and cultured overnight at 37°C. Data are shown as means with SEMs, *n* = 3; ns indicates not significant, * indicates *p* ≤ 0.05, and ** indicates *p* ≤ 0.01.

Adhesion to host cells is the first step in *S. aureus* infection. The virulence factor anchored by sortase A to bacterial cell walls is closely related to bacterial adhesion. Therefore, in this study, the effects of ECG and its analogues on the adhesion of *S. aureus* USA300 to human lung epithelial cells were analyzed. The number of *S. aureus* USA300 colonies resulting from the treatment of A549 cells in the absence of test compounds was defined as 100%. When *S. aureus* USA300-treated A549 cells received 32 µg/mL of the test compounds, the relative adhesion of the bacteria was 54.67% for C (**Figure 5A**), 14.89% for EGCG (**Figure 5B**), 46.90% for EGC (**Figure 5C**), 17.83% for ECG (**Figure 5D**) and 49.91% for EC (**Figure 5E**). ECG and EGCG showed significantly better antiadhesion effects than the other compounds, which is consistent with the above results.

### ECG and its analogues inhibit β-lactamase activity

**Figure 6:**
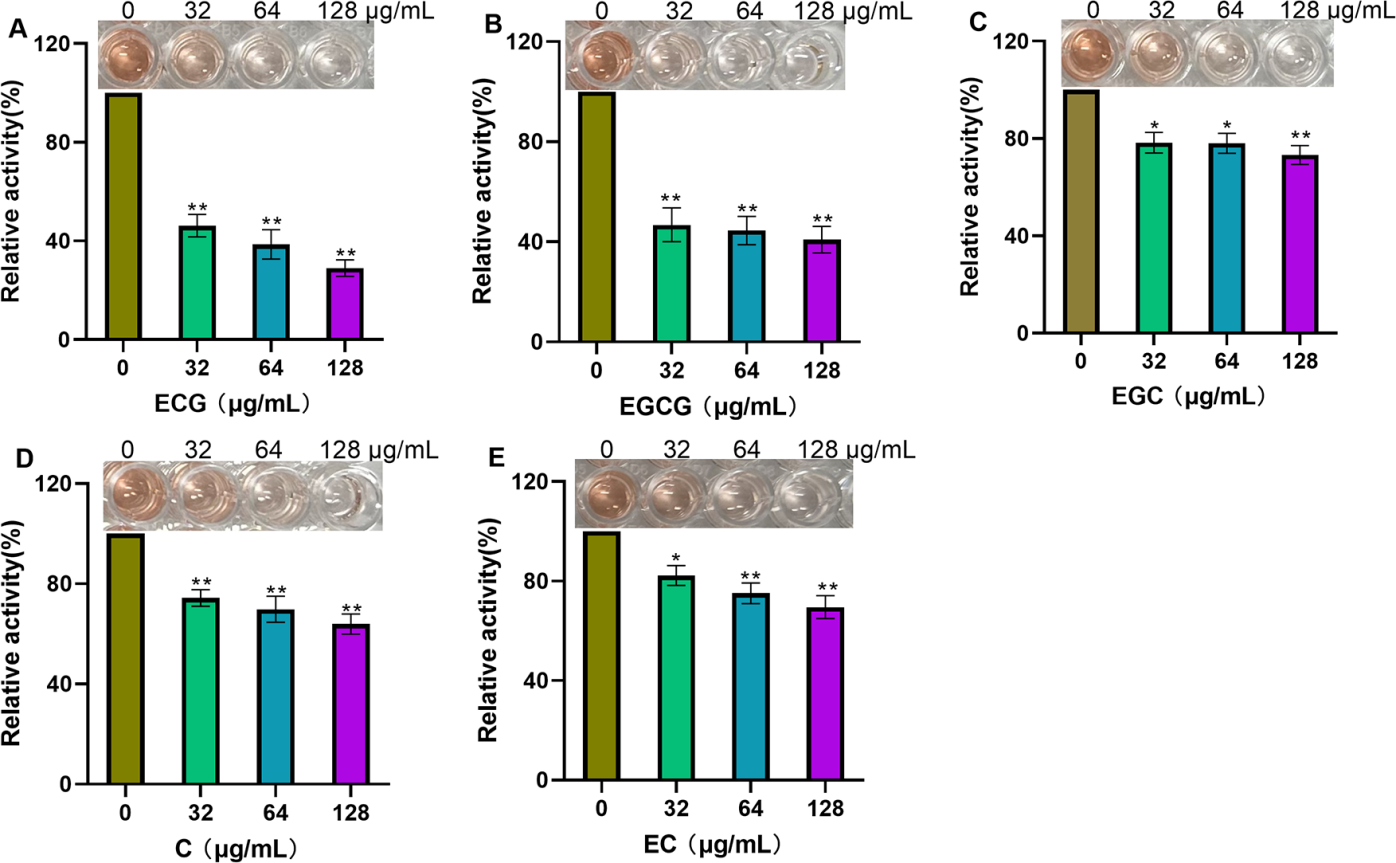
ECG and its analogues inhibit β-lactamase activity. Relative activities of β-lactamase after treatment with different concentrations of ECG **(A)**, EGCG **(B)**, EGC **(C)**, C **(D)**, and EC **(E)**. The β-lactamase protein was treated with various concentrations of the test compounds at 37°C for 30 min, and then nitrocefin was added for an additional 10 min of incubation. The OD_492_ values were measured to evaluate the inhibitory effects of these compounds on β-lactamase activity. Data are shown as means with SDs, *n* = 3; * indicates p ≤ 0.05; ** indicates *p* ≤ 0.01.

The β-lactamase secreted by *S. aureus* can decompose β-lactam antibiotics and induce bacterial tolerance. Nitrocefin is a kind of cephalosporin that is usually used to detect the activity of β-lactamases (Zhou et al., 2020), almost all β-lactamases can hydrolyze the amide bond between the carbonyl carbon and the nitrogen group on the β-lactam ring of nitrocefin, which could be judged by the color changing or detecting the OD_492_ to analyze the inhibitory effect of inhibitors on β-lactamase activity. In this assay, the samples that were not treated with ECG or its analogues were defined as 100%. When treated with 128 µg/mL test compounds, β-lactamase activity was reduced to 29.01% for ECG (**Figure 6A**), 40.85% for EGCG (**Figure 6B**), 73.24% for EGC (**Figure 6C**), 63.94% for C (**Figure 6D**) and 69.58% for EC (**Figure 6E**), indicating that ECG and its analogues inhibited β-lactamase activity.

### ECG enhances the bactericidal ability of Amp and inhibits persister formation synergistically

**Figure 7:**
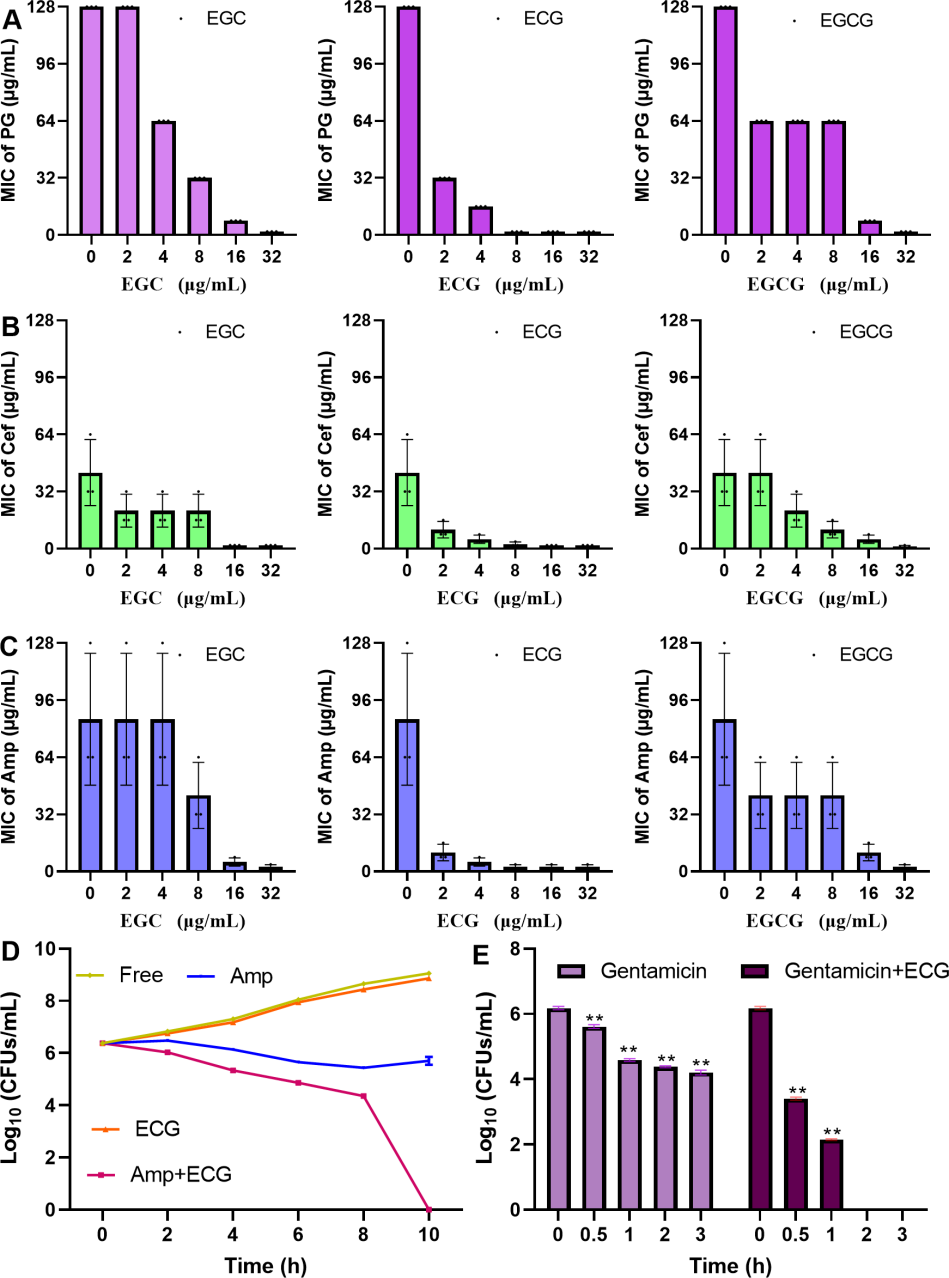
ECG enhances the bactericidal ability of Amp and inhibits persister formation synergistically. Changes in the MICs of PG **(A)**, Cef **(B)**, and Amp **(C)** upon cotreatment with EGC, ECG, and EGCG. **(D)** Logarithmic values of *S. aureus* USA300 density after treatment with ECG, Amp, and their combination. **(E)** Logarithmic values of *S. aureus* USA300 density after treatment with gentamicin with or without ECG. The MIC values of the tested antibiotics without or with ECG and its analogues were determined on the basis of CLSI methods. The *S. aureus* USA300 strain was treated with Amp, ECG, or their combination and then cultured with shaking. Samples were taken at specific time points to determine the number of clones. For the persister assay, *S. aureus* USA300 cultured overnight was treated with gentamicin with or without ECG, and colony numbers from the different samples were determined at the indicated time points. Data are shown as means with SDs, *n* = 3; ** indicates *p* ≤ 0.01.

This study evaluated the effects of ECG and its analogues on the tolerance of *S. aureus* USA300 to PG, Cef, Amp and kanamycin. When the concentrations of EGC, ECG and EGCG were 4 µg/mL, the MICs of PG and Cef were reduced by 2-, 8- and 2-fold, respectively (**Figures 7A, B**). The MIC values of Amp against *S. aureus* USA300 decreased by 16- and 2-fold when it was combined with ECG and EGCG (4 µg/mL), respectively, but cotreatment with EGC (4 µg/mL) did not change the MIC value of Amp (**Figure 7C**). The MIC value of kanamycin against *S. aureus* USA300 was greater than 256 µg/mL when kanamycin and ECG, EGCG, or EGC (32 µg/mL) was coadministered to *S. aureus* USA300 and the MIC value did not change significantly (**Table 2**), suggesting that these compounds did not synergize with kanamycin. These results indicate that ECG has excellent potential for use in combination with β-lactam antibiotics against infections caused by *S. aureus* USA300. For the synergistic sterilization assay, *S. aureus* USA300 showed similar growth trends with and without ECG treatment, but in the Amp and Amp combined with ECG treatment groups, the bacterial density gradually decreased over time. In the combination group, a more rapid downward trend was observed, and bacteria were not detected in the medium after 10 h (**Figure 7D**). The persister formation assay revealed that the bacterial density in the gentamicin treatment group decreased sharply, suggesting that the bacteria were killed quickly, but the downward trend gradually flattened, indicating that some bacteria survived as persisters; however, bacteria were not detected in the combination group after 2 h of treatment (**Figure 7E**). Taken together, these results indicate that ECG enhanced the bactericidal ability of Amp and inhibited persister formation synergistically.

**Table 2:** The MIC values of kanamycin against *S. aureus* USA300 when combined with tested compounds.

Names	MIC (µg/mL)
ECG	≥ 128
EGCG	≥ 128
EGC	≥ 128
kanamycin	> 256
Kanamycin + ECG (32 µg/mL)	> 256
Kanamycin + EGCG (32 µg/mL)	> 256
Kanamycin + EGC (32 µg/mL)	64

### ECG reduces *S. aureus* USA300-mediated cytotoxicity but does not affect the expression of its target proteins

**Figure 8:**
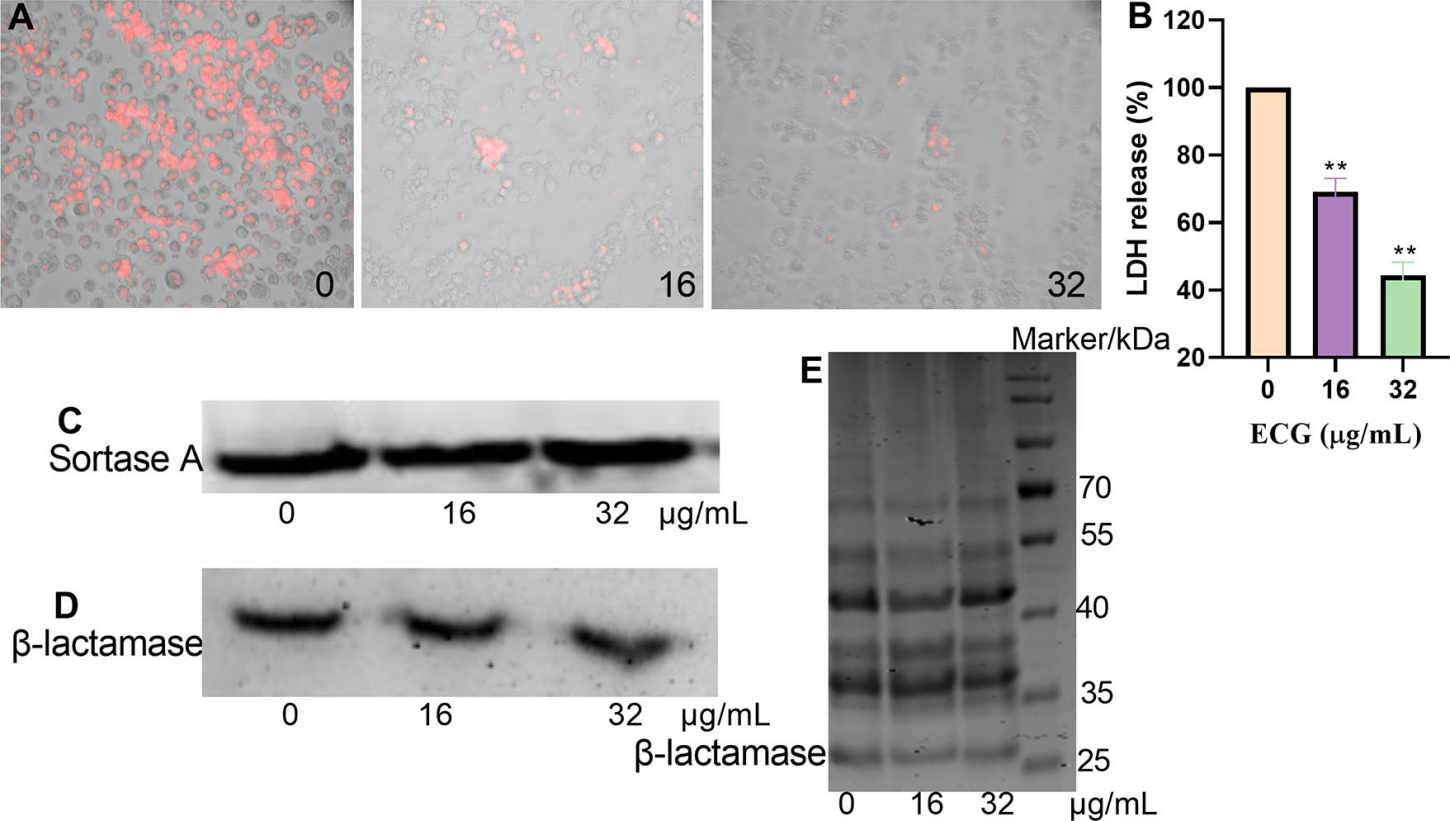
ECG reduces *S. aureus* USA300-mediated cytotoxicity but does not affect the expression of its target proteins. **(A)** Cell survival after treatment with different concentrations of ECG detected by EB staining. **(B)** LDH levels in the cell culture supernatant. RAW264.7 cells were seeded in a 96-well plate (2 × 10^4^ cells/well) and treated with *S. aureus* USA300 (MOI = 60) with or without ECG for 5 (h). The samples were centrifuged, and the supernatant was obtained. Then, an equal volume of LDH reagent was added to each well, and the mixture was incubated for 20 min in the dark. LDH levels were determined by measuring the OD_490_. The cells were stained with 50 µg/mL EB to observe their survival status. Data are shown as means with SDs, *n* = 3; ** indicates *p* ≤ 0.01. Expression levels of sortase A **(C)** and β-lactamase **(D)** and the secretion of β-lactamase into the bacterial culture supernatant **(E)**. *S. aureus* USA300 was treated with different concentrations of ECG at 37°C with shaking for 8 (h). Following centrifugation, the bacteria and supernatant were obtained, and the expression of sortase A and β-lactamase was measured via western blotting. The secretion of β-lactamase in the supernatant was evaluated via Coomassie brilliant blue staining.

*S. aureus* induces cytotoxic effects that promote the development of infection. In this study, ECG was used to evaluate the cytotoxicity mediated by *S. aureus* USA300. Red fluorescence was abundant in the *S. aureus* USA300 treatment group, indicating that most of the cells died, but the intensity of red fluorescence decreased gradually upon treatment with 16 or 32 µg/mL ECG (**Figure 8A**). In the LDH assay, the amount of LDH secreted by RAW264.7 cells treated with *S. aureus* USA300 alone was defined as 100%. When 16 or 32 µg/mL ECG was added to the samples, the amount of LDH decreased to 69.14% and 44.48%, respectively (**Figure 8B**), indicating that ECG significantly alleviated the toxicity mediated by *S. aureus* USA300 to the cells. The amounts of LDH secreted from cells treated with 32 or 64 µg/mL ECG analogues were similar to that in the DMEM treatment group (**Supplementary Table S1**), suggesting that these analogues did not exhibit cytotoxicity. Furthermore, the expression levels of sortase A and β-lactamase did not change significantly when the samples were treated with 16 or 32 µg/mL ECG (**Figures 8C, D**), and the secretion of β-lactamases in the bacterial culture supernatant was similar (**Figure 8E**), indicating that ECG did not affect the expression or secretion of β-lactamase or sortase A.

### Tyr96, Ile158, and Ile230 of β-lactamase are important for ECG binding

**Figure 9:**
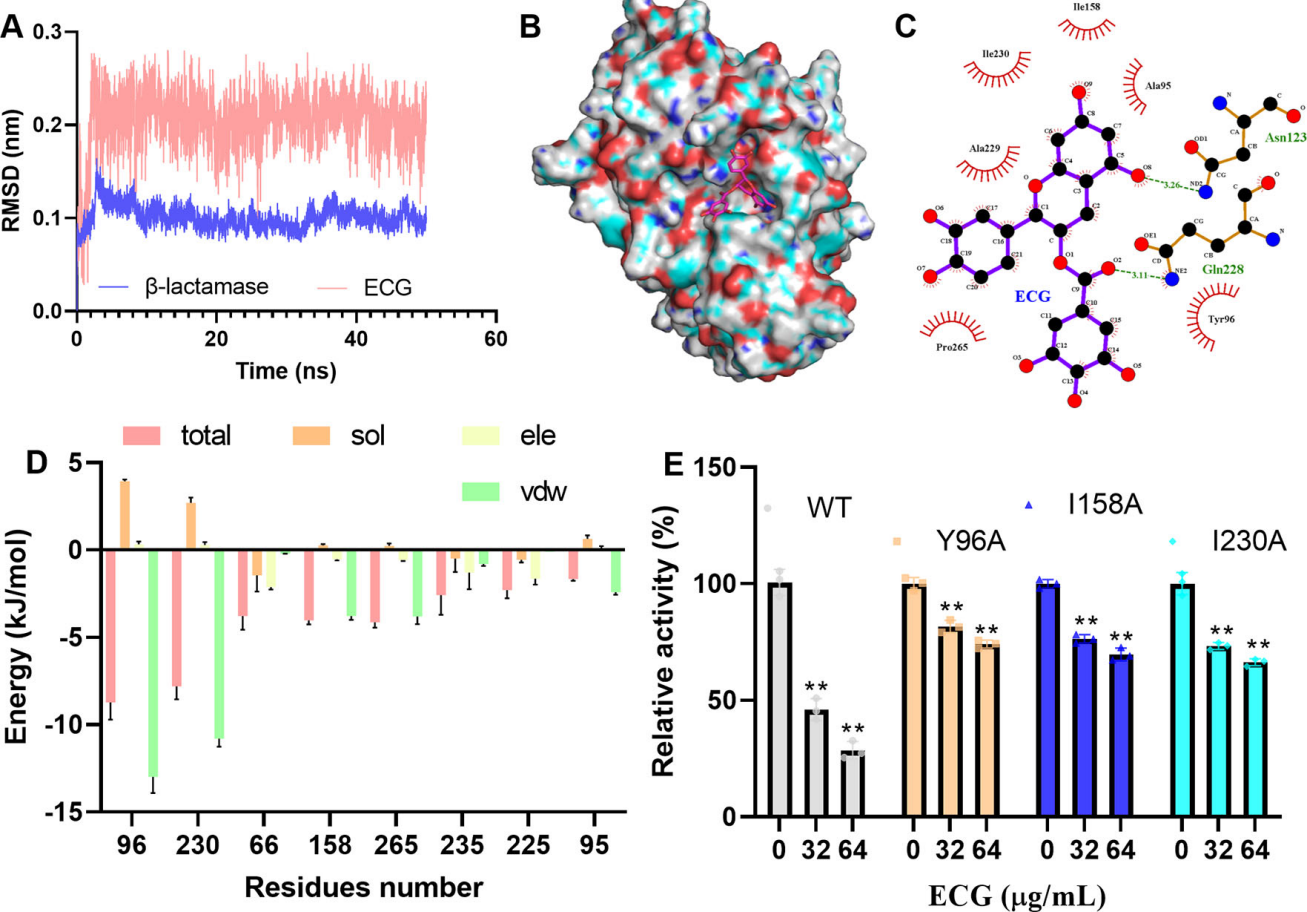
Tyr96, Ile158, and Ile230 are important for ECG binding to β-lactamase. **(A)** Fluctuations in the RMSDs during the experiment. Binding mode of ECG to β-lactamase **(B)** and the interacting residues **(C)**. **(D)** Energy contributions of the residues involved in binding. **(E)** Activities of β-lactamase and its mutants after treatment with ECG. Molecular simulations were performed by using GROMACS 2020.6, the binding free energy between β-lactamase and ECG was measured via the MMPBSA method, and the interacting residues were analyzed using LigPlus. Data are shown as means with SDs, *n* = 3; ** indicates *p* ≤ 0.01.

The β-lactamase–ECG complex was determined to be in equilibrium when the RMSD fluctuated around approximately 0.2 and 0.1 nm (**Figure 9A**). In this complex, ECG was located in the active center of the enzyme (**Figure 9B**), and binding site analysis revealed that Ile158, Ile230, Ala229, Pro265, Ala95, and Tyr96 of β-lactamase interacted with ECG, and Asn123 and Gln228 of β-lactamase formed H-bonds with ECG (**Figure 9C**). The binding free energy results revealed that Tyr96, Ile230, and Ile158 contributed more energy to this interaction, and the total free energy contributions of these residues were -8.73, -7.81, and -4.03 kJ/mol, respectively (**Figure 9D**). To identify key residues involved in ECG binding, site-directed mutagenesis of β-lactamase was performed, and the results revealed that the inhibitory effects of ECG against the Y96A, I158A, and I230A mutants were much lower than that against the WT protein (**Figure 9E**), suggesting that these residues are critical for ECG binding.

### ECG combined with Amp protects mice from *S. aureus* USA300 infection

**Figure 10:**
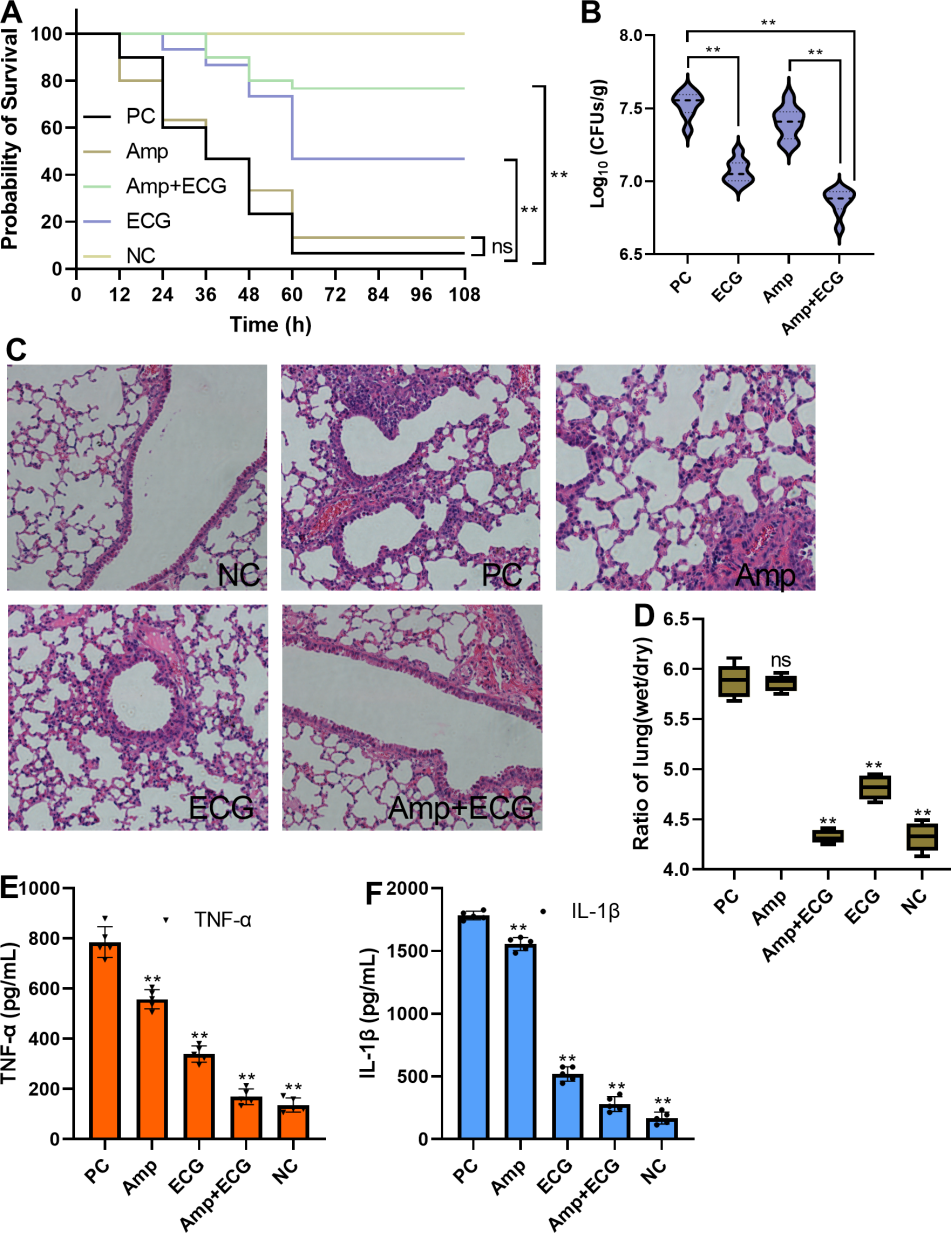
ECG combined with Amp protects mice from *S. aureus* USA300 infection. **(A)** Survival of the mice in the different treatment groups, *n* = 30. **(B)** Bacterial loads in the lungs of the mice in the different treatment groups, *n* = 8. **(C)** Degree of pathological injury in each of the different treatment groups. **(D)** Lung weight ratio and level of inflammation in the lungs **(E, F)** in different treatment groups. C57BL/6J mice were intranasally inoculated with *S. aureus* USA300 (5 × 10^8^ CFUs/mouse) to establish a pneumonia model. The mice were treated with 50 mg/kg ECG, Amp, or their combination every 12 (h). To evaluate the survival rate, the mice were monitored at the specified time points. To evaluate the other indicators, lung tissues were obtained after the mice were euthanized. The bacterial loads of the lung tissues from the different groups were evaluated by analyzing the numbers of clones, and inflammation, pulmonary edema, and tissue damage were assessed by detecting the levels of cytokines, the weight ratio, and HE staining, respectively. Data are shown as means with SDs; *n* = 5; ns indicates not significant; ** indicates *p* ≤ 0.01.

Mice in the PC and Amp treatment groups died after 12 h, and the survival ratios of the two groups were 6.67% and 13.33%, respectively. However, in the ECG and combination groups, the mice died at 24 or 36 h, and the survival ratios reached 46.67% and 76.67%, respectively (**Figure 10A**). In addition, the number of *S. aureus* USA300 colonies in the ECG and ECG combined with the Amp treatment groups was significantly lower than that in the PC and Amp treatment groups (**Figure 10B**). Additionally, the lung tissues from the PC and Amp treatment groups exhibited inflammatory factor infiltration and the alveolar structures were destroyed compared with those in the NC group, whereas these manifestations were alleviated in the samples from the ECG and ECG combined with Amp groups (**Figure 10C**). Compared with that in the PC group, the wet/dry lung weight ratios from the ECG and ECG combined with Amp treatment groups were significantly lower, but Amp treatment alone did not affect this ratio (**Figure 10D**). As important indicators of inflammation level, TNF-α and IL-1β are often used to quantitatively evaluate the inflammation level of cells or tissue (Li et al., 2019; Wang et al., 2022). *S. aureus* infection is usually accompanied by an excessive inflammatory response, accompanied by an excessive release of TNF-α and IL-1β (Ghosh et al., 2023; Ghosh and Bishayi, 2024); the levels of TNF-α and IL-1β here in the samples from the ECG and ECG with Amp groups were significantly lower than those in the other groups, and Amp treatment alone slightly reduced the levels of these cytokines (**Figures 10E, F**). These results suggest that ECG alone or in combination with Amp could delay mouse death, improve mouse survival, weaken the colonization ability of bacteria, and relieve edema and inflammation in the lungs.

## Discussion

Sortase A is the most important member of the sortase family and anchors more than 20 different surface proteins to the bacterial cell wall (Geoghegan and Foster, 2017). The biological functions of some of these surface proteins, such as fibronectin-binding proteins that influence adhesion and biofilm formation, have been characterized (Kim et al., 2018; Foster, 2019; Xu et al., 2024). Chalcone has been reported to reduce the pathogenicity of *Listeria monocytogenes* by inhibiting sortase A activity by occupying the active center of sortase A (Li et al., 2016). Astilbin and trans-chalcone have been shown to inactivate *Streptococcus mutans* sortase A and reduce their formation of biofilms (Wallock-Richards et al., 2015; Wang et al., 2019). Wogonin was found to attenuate the pathogenicity of *Streptococcus pneumoniae* by inhibiting pneumolysin and sortase A, but the mechanism has not been elucidated (Gu et al., 2023). Here we found that ECG and its analogues bind directly to *S. aureus* sortase A and significantly reduce its activity; consequently, the *S. aureus* biofilm formation and adhesive abilities were inhibited, possibly resulting from the inactivation of sortase A, although other potential mechanisms maybe exist.

The crystal structure of sortase A and its substrate complex was reported in 2004 (PDB ID: 1T2W) (Zong et al., 2004). The substrate LPETG is located in the enzyme’s catalytic center, with its N-terminus located around hydrophobic residues Ile158, Ile199, Val201, Val 168, and Leu169 and its C-terminus pointing to the narrow opening of the active site, wherein the key active site residues His120, Cys184, and Arg197 are positioned around the substrate peptide (Zong et al., 2004). In this study, the crystal structure of sortase A (PDB ID: 6R1V) was used for molecular docking, and it was found that ECG binds to the active pocket and that Leu169, Val168, Lys173, Asp170, Ile182, and Thr180 in sortase A had greater contributions to the binding free energy. Additionally, the inhibitory effects of ECG against the V168A, L169A, and K173A mutants decreased significantly, as these residues were located on the active center of the enzyme.

β-lactam antibiotics have played an important role in combating bacterial infections since the application of penicillin in the treatment of *S. aureus* infection. Unfortunately, the emergence and diversification of β-lactamases have posed challenges to the control of bacterial infections (Chen and Herzberg, 2001; Mlynarczyk-Bonikowska et al., 2022), limiting the clinical efficacy of all currently available β-lactam antibiotics. Therefore, it is important to identify antibiotic adjuvants, especially natural compounds with no or little antibacterial activity, to improve the sustainability of these types of antibiotics in clinical applications. To date, some natural compounds used as adjuvants have been shown to reduce pathogenic bacterial virulence, such as plumbagin, which binds to the catalytic pocket of monooxygenase to reduce the resistance of tet(X3)/tet(X4)-positive bacteria to tetracycline (Xu et al., 2022); thymol nanoemulsion, which reverses colistin resistance in *Salmonella enterica serovar Typhimurium (* Sheng et al., 2023); and fisetin, which attenuates meropenem resistance in NDM-1-producing *E. coli* by targeting metallo-β-lactamases (Guo et al., 2022). This study revealed that ECG and its analogues restored the sensitivity of bacteria to Amp, Cef, and PG by inhibiting β-lactamase activity. ECG improved the bactericidal ability of Amp and inhibited the formation of persisters. ECG alone or in combination with Amp *in vivo* had a systemic protective effect in the *S. aureus* pneumonia model. These results suggest that ECG has the potential to be developed as an adjuvant for β-lactam antibiotics in the future to improve the sustainability of antibiotic applications.

O Herzberg reported the crystal structure of the *S. aureus* β-lactamase (PDB ID: 3BLM), which is composed of two domains, and the catalytic center is located at the interface between the two domains. Many residues are found in the catalytic pocket, including but not limited to Tyr105, Glu166, Leu169, Asn170, Lys73, Ser70, Ile239, Ser130, Lys234, and Tyr165 (Herzberg, 1991). Crystal structures of β-lactamases with their ligands were subsequently reported (PDB IDs: 1BLC and 6WGR), and both ligands were located in the catalytic center (Chen and Herzberg, 1992). Here the β-lactamase crystal structure 6WGR was used to perform molecular docking, and it was found that ECG bound to the catalytic pocket of β-lactamase. The calculated binding free energy and mutagenesis analysis results revealed that Tyr96, Ile158, and Ile230 are important for ECG binding. We aligned the 6WGR and 3BLM structures and found that Tyr96, Ile158, and Ile230 in 6WGR corresponded to Tyr105, Ile167, and Ile239 in 3BLM, which are the constituent residues of the active pocket.

## Conclusion

This study revealed that green tea bioactive ingredients, especially ECG, target *S. aureus* sortase A and β-lactamase simultaneously to alleviate the pathogenicity of *S. aureus*. By interacting with sortase A, ECG decreased *S. aureus* biofilm formation and the adhesion of this bacteria to host cells. ECG also bound to β-lactamase to restore the susceptibility of this bacteria to β-lactam antibiotics, improve the bactericidal ability of Amp, and reduce the formation of persisters. ECG alleviated the cytotoxicity mediated by *S. aureus* USA300 without affecting the expression of sortase A or β-lactamase and protected mice from *S. aureus* USA300 infection comprehensively. These results support the potential of the development of ECG or its analogues as anti-*S. aureus* infection agents.

## Data Availability

The raw data supporting the conclusions of this article will be made available by the authors, without undue reservation.
