# Supplementary material for: Epicatechin gallate and its analogues interact with sortase A and β-lactamase to suppress Staphylococcus aureus virulence
Source: Front Cell Infect Microbiol. 2025 Mar 25;15:1537564. doi: 10.3389/fcimb.2025.1537564 (PMC11975897; doi:10.3389/fcimb.2025.1537564)
Supplement: Supplementary file 1 [file Table1.docx]

***Supplementary materials for***

**Epicatechin gallate and its analogues interact with Sortase A and β-lactamase to suppress *Staphylococcus aureus* virulence**

Fei Teng^a#^, Lihui Wang^a#^, Jingyao Wen^b^, Zizeng Tian^b^, Guizhen Wang^b#^, Liping Peng^a#^

^a^Department of Respiratory Medicine, The First Hospital of Jilin University, Changchun 130021, China

^b^College of Biological and Food Engineering, Jilin Engineering Normal University, Changchun, 130052, China

**^#^**These authors contribute equally to this work

***Correspondence:** Guizhen Wang [(wanggz@jlenu.edu.cn);](mailto:(wanggz@jlenu.edu.cn);)

Liping Peng ([penglp@jlu.edu.cn](mailto:penglp@jlu.edu.cn))

Table S1 The cytotoxicity of the tested compounds against RAW cells

| **Compounds name** | **LDH levels (%)** | |
| --- | --- | --- |
|  | **32 µg/mL** | **64 µg/mL** |
| ECG | 8.81 ± 1.33 | 9.54 ± 0.1 |
| EGCG | 8.68 ± 1.65 | 9.07 ± 2.63 |
| C | 7.20 ± 1.33 | 9.80 ± 1.88 |
| EC | 6.45 ± 0.96 | 8.06 ± 1.28 |
| EGC | 6.29 ± 1.00 | 6.97 ± 1.02 |
| DMEM | 5.63 ± 0.11 | |
| 0.1% Triton X-100 | 100.04 ± 4.63 | |
